# Retraction: Homonuclear bond activation using a stable *N*,*N*′-diamidocarbene

**DOI:** 10.1039/c5sc90019k

**Published:** 2015-04-21

**Authors:** Kelly M. Wiggins, Jonathan P. Moerdyk, Christopher W. Bielawski

**Affiliations:** a Department of Chemistry and Biochemistry , The University of Texas at Austin , 1 University Station A1590 , Austin , 78712 , TX , USA . Email: bielawski@cm.utexas.edu

## Abstract

Retraction of ‘Homonuclear bond activation using a stable *N*,*N*′-diamidocarbene’ by Kelly M. Wiggins *et al.*, *Chem. Sci.*, 2012, **3**, 2986–2992.



## 

The Royal Society of Chemistry hereby wholly retracts this *Chemical Science* article with the agreement of Christopher W. Bielawski, Jonathan P. Moerdyk, and Kelly M. Wiggins due to data fabrication as detailed below. This retraction supersedes the information provided in the Expression of Concern related to this article.

The Royal Society of Chemistry has been contacted by the corresponding author of this article and the Research Integrity Officer at The University of Texas at Austin regarding concerns of scientific misconduct affecting this article. The Research Integrity Officer has informed us that an investigation to ascertain the validity of the work reported has found that scientific misconduct by one of the article's co-authors has taken place as follows: Scheme 5 and the last paragraph under the heading “Disulfides” in the Results and Discussion section discusses fabricated data. The NMR spectra figures on page S36 were also fabricated. The synthesis of *S*-methyl thiobenzoate and the compound denoted 7a* did not occur as reported. No reaction occurred and compound 8a was isolated. The characterization data for the two reported compounds was fabricated from other spectra. The signing authors would like to apologise for this and any consequent inconvenience to authors and readers.

Signed: Christopher W. Bielawski, Jonathan P. Moerdyk, and Kelly M. Wiggins, March 2015.

Retraction endorsed by May Copsey, Executive Editor, *Chemical Science*.

